# Packaging Technology for an Implantable Inner Ear MEMS Microphone

**DOI:** 10.3390/s19204487

**Published:** 2019-10-16

**Authors:** Lukas Prochazka, Alexander Huber, Ivo Dobrev, Francesca Harris, Adrian Dalbert, Christof Röösli, Dominik Obrist, Flurin Pfiffner

**Affiliations:** 1 Head and Neck Surgery, Department of Otorhinolaryngology, University Hospital Zurich, 8091 Zurich, Switzerland; 2 University of Zürich, 8091 Zurich, Switzerland; 3 Cochlear Technology Centre, 2800 Mechelen, Belgium; 4 ARTORG Center, University of Bern, 3010 Bern, Switzerland

**Keywords:** cochlear implant, implantable microphone, intracochlear acoustic receiver, MEMS microphone, electronic packaging, lumped element model, anechoic water tank, inner ear

## Abstract

Current cochlear implant (CI) systems provide substantial benefits for patients with severe hearing loss. However, they do not allow for 24/7 hearing, mainly due to the external parts that cannot be worn in all everyday situations. One of the key missing parts for a totally implantable CI (TICI) is the microphone, which thus far has not been implantable. The goal of the current project was to develop a concept for a packaging technology for state-of-the-art microelectromechanical systems (MEMS) microphones that record the liquid-borne sound inside the inner ear (cochlea) as a microphone signal input for a TICI. The packaging concept incorporates requirements, such as biocompatibility, long-term hermeticity, a high sensing performance and a form factor that allows sensing inside the human cochlea and full integration into the existing CI electrode array. The present paper (1) describes the sensor packaging concept and the corresponding numerical and experimental design verification process and (2) gives insight into new engineering solutions for sensor packaging. Overall, a packaging concept was developed that enables MEMS microphone technology to be used for a TICI system.

## 1. Introduction

Unlike conventional hearing aids that amplify sound at the outer ear, a cochlear implant (CI) bypasses the damaged sensory components of the ear to provide electrical stimulation directly to the nerves within the cochlea (inner ear). Nowadays, CI systems are partially implantable devices utilizing an external (behind the ear) sound processor that incorporates the microphone components. The processed sound information is transmitted to the implanted CI components via transcutaneous magnetic induction.

Despite CIs being a success story, there remain substantial needs that cannot be addressed with partially implantable CI systems. The external parts are susceptible to humidity, moisture, dust and direct mechanical impact. They are uncomfortable for sleeping and can cause stigma. A totally implantable CI system (TICI) would provide recipients with significant safety benefits associated with 24/7 hearing and overall improved quality of life. So far, a TICI system does not exist on the market, mainly due to the difficulty of providing an implantable microphone technology that meets stringent requirements for performance, safety and cost efficiency.

Two commercially available implantable microphones exist as part of totally implantable middle ear implants (Esteem^®^, Envoy Medical Corporation, White Bear Lake, MN, USA and Carina™ Cochlear Ltd., Sydney, Australia) [1,2,3]. The Esteem system uses piezoelectric technology for the acoustic sensor that detects the vibration of the middle ear ossicles. The system requires a complex surgical intervention and suffers from high surgical complication rates [4,5]. The implantable microphone of the Carina™ system is a large subcutaneous condenser microphone, implanted on the skull in a relatively muscle-free area behind the patient’s ear. It is designed to detect airborne sound through the overlying skin layer. Despite further optimization of the implantable microphone of the Carina™ system, its integration in a commercial TICI system has still not yet been achieved.

Several research groups are working on implantable vibration sensors (accelerometers and force sensors) for sensing the middle ear motion [6]. They are based on different transduction principles, including optical, piezoelectric, piezoresistive and capacitive means. All concepts are still under development and not yet commercially available.

Another approach for an implantable microphone is to measure the sound pressure signal in the perilymph of the inner ear. Such a solution, which we call the intracochlear acoustic receiver (ICAR), would suit CI-recipients who have an intact middle ear. The sound recorded by the ICAR incorporates the natural transformation and processing of sound by the outer and middle ear that is associated with directivity cues and amplification. Compared to a subcutaneous microphone, an ICAR supposed to be less sensitive to skin contact and direct mechanical impact An ICAR that is integrated into the existing electrode array of the CI would not require additional surgical interventions, which is the main drawback of middle ear microphones.

Custom-made or commercial miniature fibre-optic pressure sensors are used by several research groups for intracochlear sound pressure measurements in animals and human temporal bones with the aim of investigating hearing physiology [7,8,9]. Using optical transduction for an implantable ICAR is difficult to realize with current light source technology, mainly because of power restriction and difficult system integration. Recently, attempts have been made to use piezo-electric material to build implantable ICARs of different geometries (stripe, beam and membrane) and based on different sensing principles (deformation, displacement and resonance) [10,11,12]. Proof-of-concept studies could demonstrate the feasibility of sensing the intracochlear sound pressure in animal experiments using piezoelectric technology but the sensing performance does not meet the specifications for a microphone of a TICI system. In addition, requirements relevant for an implantable microphone such as long-term biocompatibility, electromagnetic interference immunity and low power consumption are not considered in these studies.

We aim to develop an ICAR which is specifically designed to meet all requirements for an implantable microphone and in particular, to enable its integration into the existing electrode array of the CI. Our approach is to use commercial high-end microelectromechanical systems (MEMS) microphone (MEMS MIC) technology (capacitive or piezoelectric) which we install in a customized long-term hermetic titanium enclosure, to provide biocompatibility and to enable sensing in the perilymph space of the inner ear. The main objectives during the design and fabrication of the protecting structure are (1) to optimize its size, geometry and layout with regard to a simple surgical intervention and (2) to minimize the loss in sensing performance of the MEMS MIC caused by the hermetic encapsulation. 

A first non-implantable MEMS Condenser Microphone-Based ICAR prototype became a dedicated research tool for intracochlear sound pressure measurements in animal and human temporal bones performed in our lab. The sensor and the corresponding application are described in Pfiffner et al., 2016 [13] and Péus et al., 2017 [14]. In another study, acute in vivo animal experiments were performed to verify the surgical intervention required for the implantation of the ICAR. The partially implantable prototype of the ICAR used for these experiments and the outcome of the study are published in Pfiffner et al., 2018 [15].

The present paper describes the packaging concept and the design study that was conducted to verify the sensing performance of the proposed ICAR of a TICI system. The study comprised theoretical and experimental investigations using a prototype sensor that exhibits performance relevant features of the packaging structure of the MEMS MIC. In addition, the design and fabrication approach of an implantable ICAR prototype is discussed that is foreseen to be used for chronic in vivo animal experiments.

## 2. Materials and Methods

### 2.1. Design Requirements

Simplicity and a low complication rate of surgical intervention are important criteria in the design process of implantable devices. Therefore, the ICAR is designed to be implanted into the scala tympani (ST) of the inner ear as an integral part of the existing CI electrode. Such a TICI system requires only one point of surgical access and a similar surgical procedure as that already established for existing CI systems. Sensor location in the scala vestibuli (SV) would be beneficial from a hydrodynamic point of view (higher sound pressure) but additional, complicated surgical access would be necessary.

**Figure 1:**
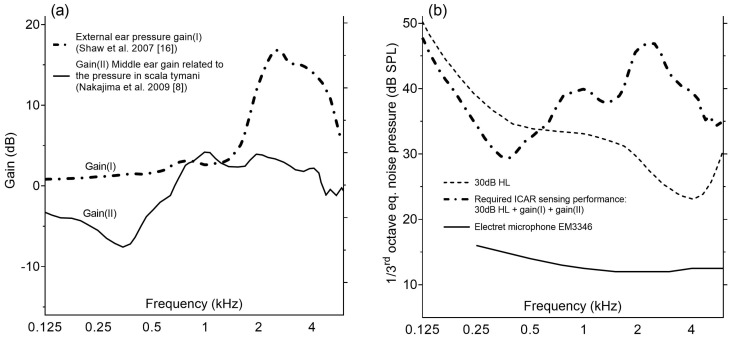
(**a**) Outer ear pressure gain(I) (zero incident angle) from Shaw et al., 2007 [16] and the middle ear pressure gain(II) in the basal part of the scala tympani (ST) from Nakajima et al., 2009 [8]. (**b**) Required sensing performance of the intracochlear acoustic receiver (ICAR) implanted into the ST and expressed as a one-third octave equivalent input noise level. The maximum allowable equivalent input noise of the ICAR is the sum of the 30 dB hearing level (HL) line and the outer and middle ear pressure gain. For the purpose of comparison, the noise level of an electret microphone EM3346 typically used for hearing aid devices is also shown.

As for a typical hearing aid microphone, the operating bandwidth of the ICAR is from 200 Hz–6 kHz. The sensing performance of the ICAR is expressed as the equivalent input noise (EIN) level in one-third octaves. The value is calculated from the self-noise (voltage) and the sensitivity (frequency response) of the receiver. The maximum allowable EIN of an ICAR implanted in the ST is defined as the 30 decibel hearing level (dB HL) curve plus the pressure gain caused by the outer and middle ear (Figure 1). The unit dB HL relates the measured sound to the quietest sound that a young healthy individual ought to be able to hear (zero reference level in audiometry, DIN EN ISO 389-1:2018-06). The hearing capability of humans is regarded as sufficiently good if the hearing threshold does not exceed 30 dB HL. The outer and middle ear gain both show a strong frequency dependence (Figure 1a). The former gain originates from resonance in the ear canal (semi-open pipe) [16] and the latter one from the middle ear, which efficiently converts air borne sound into liquid borne sound in the inner ear (impedance matching) [8].

The power consumption of the ICAR is restricted to 100 μW (signal processing unit not included). This low value is dictated by the limited capacity of an implantable battery (or supercapacitor) due to more restricted space conditions compared to an external sound processor [17].

The ICAR is designed such that it can remain implanted in the body for several decades (>70 years). Hence, all components of the ICAR must be long-term biocompatible or hermetically sealed in an appropriate packaging structure. The sensing performance must be stable over the life span of the ICAR. In addition, all electronic components which carry a DC current or voltage must be electrically insulated from the body. 

### 2.2. Packaging Concept

The core of the ICAR is an MEMS MIC that can be of any transduction principle (capacitive, piezo-electric and resistive or optical) as long as requirements for size, sensing performance and power consumption are met. State-of-the-art MEMS MICs are commercially available devices that are developed mainly for the telecommunication market (smart phones, cameras, etc.). This target market (> billion units per year) justifies huge investments in MEMS MIC development, which is an effort that is expected to continue. Using this off-the-shelf high-end sensing technology is one of the key benefits of the present sensor concept. 

**Figure 2:**
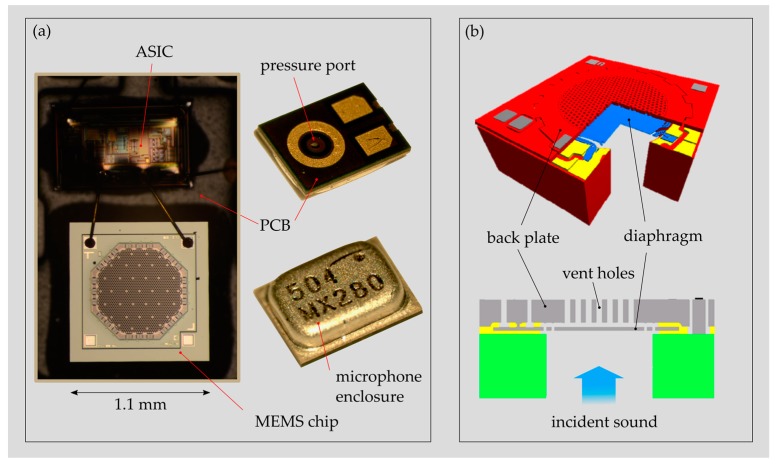
(**a**) Typical packaging of a commercially available analogue MEMS condenser microphone (MEMS CMIC) (ADMP 504, Analog Devices Inc., Norwood, MA, USA) with the MEMS chip and the ASIC mounted on a printed circuit board (PCB) and enclosed with a metallic housing. (**b**) Schematic drawing of a MEMS CMIC with a typical configuration of the back plate and diaphragm (adapted from Reference [20]).

Nowadays, most of the commercial MEMS MICs are MEMS condenser microphones (MEMS CMICs) that are based on the capacitive transduction principle. They provide the best performance/size ratio, low power consumption and a very stable unit-to-unit performance [18,19]. MEMS CMICs optimized for the hearing aid market (e.g., Puma-Series, Sonion Inc., Roskilde, Denmark) measure only 8.2 mm^3^ (3.35 mm × 2.5 mm × 0.98 mm), provide a noise level (EIN) of 25 dB(A) sound pressure level (SPL) and a battery drain of 31 μA at 1 V supply voltage. The microphone packaging contains the MEMS chip (motor) and the corresponding application-specific integrated circuit (ASIC) unit, providing amplification and signal conditioning (Figure 2a). Both parts are mounted on a rigid printed circuit board (PCB) and enclosed with a metallic housing providing protection, electromagnetic interference (EMI) shielding and a reference chamber. The MEMS chip, which is usually less than 1 mm^3^ large, exhibits two freestanding, parallel plates suspended on a single-crystal silicon (SCS) structure (Figure 2b). One plate represents a compliant diaphragm that starts to vibrate with incoming sound and the opposite one, the back plate, is the rigid plate of the capacitor. 

Commercially available MEMS CMICs are neither biocompatible nor can they operate in a liquid environment, such as in the perilymph of the inner ear. Despite their small size, they are far too big to be inserted directly into the human cochlea. Therefore, a packaging solution that is customized to the present application is required. In the following description of the packaging concept, characteristic numbers are stated that are justified by theoretical and experimental studies discussed later in this text. 

**Figure 3:**
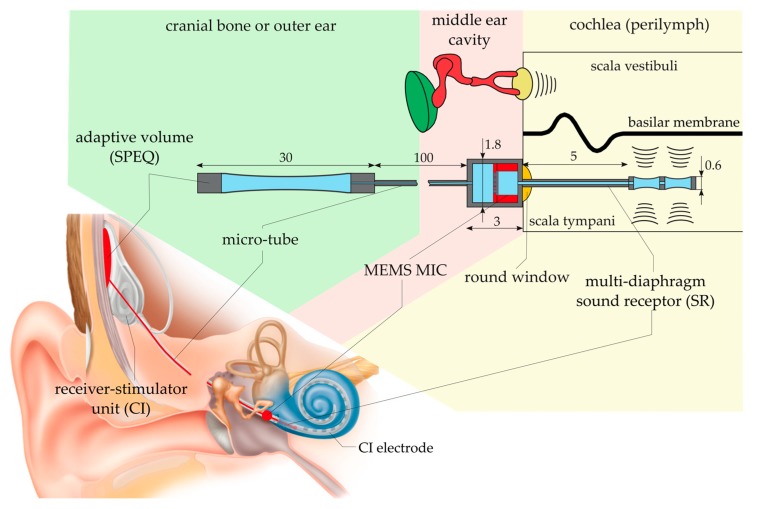
Schematic illustration of the implantable packaging structure protecting an off-the-shelf MEMS microphone (MEMS MIC) against body liquid and vice versa. The location of the individual components of the structure in the human body is indicated schematically and in an anatomical illustration of the human ear. In the latter representation, the intracochlear acoustic receiver (ICAR) is shown as an integral part of a cochlea implant system.

The basic idea of the present microphone packaging is to enclose the MEMS chip and the corresponding ASIC in a biocompatible housing that also seals the electronic devices against the liquid environment. We presume a considerably longer lifetime of a MEMS CMIC protected by a long-term hermetic enclosure than of a device in direct contact with surrounding conditions (particle contamination and moisture). To enable sound energy to enter the packaging structure, a thin diaphragm, called the protective diaphragm (PD), is integrated into the protective structure. The upper limit of the operating frequency range of a sealed microphone is determined by the resonance frequency of the PD, which is below 10 kHz in water (Section 3.1 and Section 3.2.1). Four 0.6 mm large PDs instead of one large PD are used to maximize both the sensitivity and the bandwidth of the sealed MEMS CMIC (Section 3.2.1). In order to account for the tiny dimensions of the human cochlea, the MEMS CMIC unit is kept outside the cochlea in the middle ear cavity adjacent to the round window (RW) with the PD’s immerged in the inner ear fluid (Figure 3). 

Due to an increasing sound pressure with increasing distance from the RW and a higher risk of ossification within the basal part of the ST after CI implantation, the optimum sensing location for intracochlear sound pressure is as far upstream as possible in the ST [21,22]. Currently, pressure sensing approximately 5 mm upstream of the RW is foreseen. Positions further upstream are mainly restricted by the narrowing lumen and the increasing curvature of the cochlea channel in apical direction. For pressure sensing upstream of the RW, the PDs are installed within the tip region of a tube-like structure, inserted into the ST through the RW and attached to the pressure port of the MEMS CMIC at the other end (Figure 3). This part of the packaging structure is called the sound receptor (SR). 

Microphones are designed to capture pressure variations below 1 mPa. To prevent excess loading of the very compliant sensing diaphragms due to static pressure variations (e.g., atmospheric pressure variations) that are orders of magnitude higher than typical sound pressure levels, a system for static pressure equalization (SPEQ) is required. In an MEMS MIC, this is typically realized by tiny vent holes within the sensing diaphragm that separates the back chamber from the surroundings. An ICAR that is hermetically encapsulated requires an SPEQ system that works without an open interface to the surroundings. One possible system which works completely passive is a larger adaptive volume located in the mastoid (external part of the temporal bone) similar to the receiver-stimulator unit of the CI and pneumatically interconnected with the microphone housing via a flexible tubing (Section 2.5.3). For internal volume adaptation, large, highly compliant diaphragms deflect, dependent on the ambient pressure and adapt the internal volume upon pressure equalization (Section 2.3.2). Such an SPEQ system has a limited operating range which is designed to cover ambient pressure variations from 700 mbar to 1200 mbar (e.g., due to weather changes, flight travel, etc.). Nevertheless, using a passive system a certain drop in sensitivity prevails, even within the covered pressure range, since full pressure equalization can only be achieved for small variations from the internal pressure (Section 3.2.2).

Titanium is chosen as the material for the whole packaging structure consisting of the SR, the MEMS MIC housing and the SPEQ system. This choice results from four design requirements: long-term biocompatibility, long-term hermeticity, manufacturability and EMI compatibility. For electrical insulation purposes, the whole titanium structure of the ICAR should be coated with a 10–100 nm thick parylene film. Parylene is biocompatible, has excellent mechanical and electrical insulation properties and can be applied pinhole-free on any geometry as a sub-micron protective film. Because of the low elastic compliance of parylene (Young’s modulus 4 GPa vs. 110 GPa of titanium), we do not expect a significant influence of the coating on the compliance of the PDs. Therefore, the protective coating is not further considered in the performance analysis of the ICAR (Section 2.3.1 and Section 3.2.1).

### 2.3. Theoretical Verification of the Packaging Concept

For the theoretical verification of the packaging concept, the sensor system was split into a receiver unit containing the microphone housing and the SR and the system for SPEQ. The feasibility of the receiver unit was investigated using a theoretical model and by experiments, whereas the SPEQ system was verified by theoretical means. 

#### 2.3.1. Theoretical Model of an ICAR Sealed with Multiple PDs

A lumped element model (LEM) representing an MEMS CMIC sealed with multiple PDs was used to define the dimensions of the ICAR packaging structure that conforms with the requirements for the sensing performance (sensitivity and bandwidth) and geometrical constraints. The model was validated by experimental tests in air and in water using a simple prototype sensor representing the packaging concept (Section 2.4). LEMs are based on the analogy between electrical, mechanical and acoustic systems that holds if the largest geometrical dimension of the system is much smaller than the wavelength of a sound wave in the corresponding fluid or solid [23]. If this is fulfilled, spatial variations of physical quantities can be neglected and no wave propagation phenomena occur. In an LEM of an acousto-mechanical system, all elements, such as the diaphragm, cavities and tubing, are expressed as lumped parameters representing impedances of an equivalent electrical circuit. The sound pressure *p* (or force) is equivalent to the voltage and the volume flow *q* (or velocity) to the current of an electrical system. In the case of an ICAR, only sound at frequencies below 10 kHz is considered. The corresponding minimum wavelength in air is 34 mm and 150 mm in water. The largest geometrical dimension in an ICAR is the micro-channel of the SR, which should not exceed a length of 5 mm. Therefore, using a LEM for the present application is valid.

**Figure 4:**
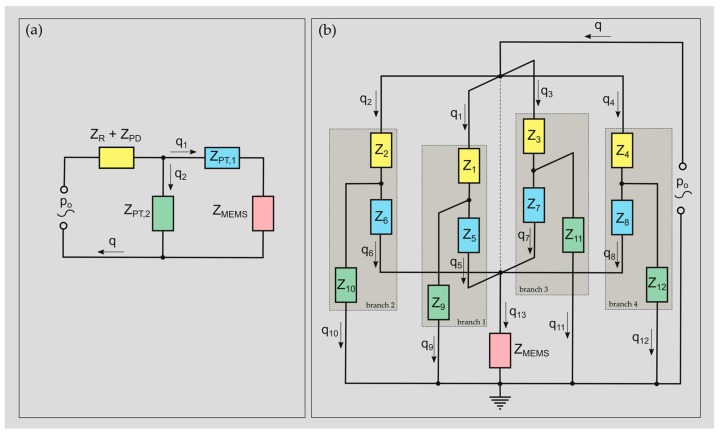
(**a**) Simplified equivalent electrical circuit representing the lumped element model (LEM) of an MEMS microphone (MEMS MIC) sealed with one protective diaphragm (PD) and (**b**) with up to four PDs. *Z*_1_ … *Z*_4_: acoustic impedance of the PD and its interaction with the surroundings; *Z*_5_ … *Z*_8_: dissipation and inertia caused by the pneumatic tubing system between the PD and MEMS diaphragm; *Z*_9_ *… Z*_12_: acoustic stiffness of the pneumatic tubing system between the PD and MEMS diaphragm; *Z_MEMS_*: acoustic impedance of the (unsealed) MEMS MIC without considering the inlet channel (pressure port).

**Figure A1:**
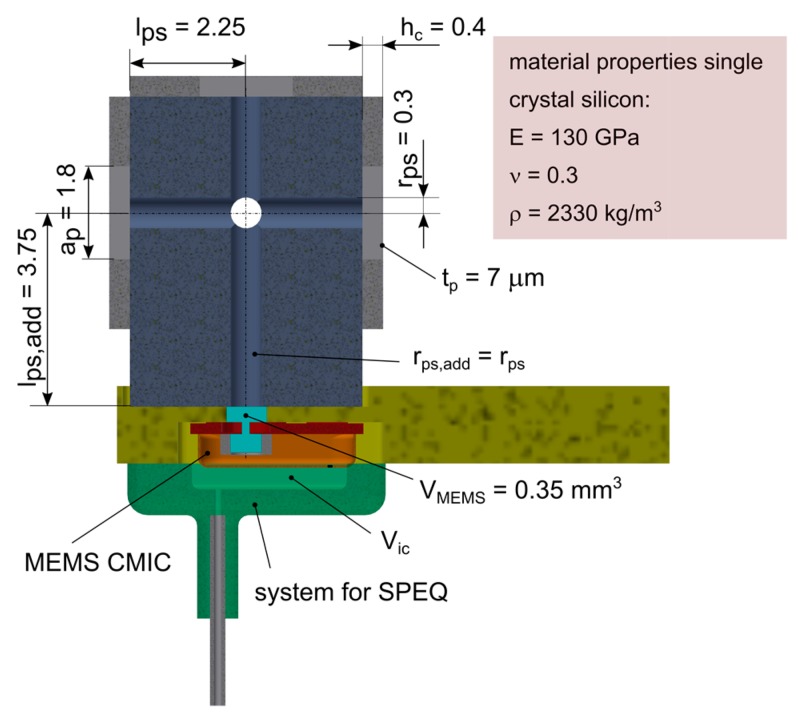
Input parameters in mm of the lumped element model (LEM) characterizing only the protective diaphragm(s) and the corresponding pneumatic tubing system. Input parameters representing the MEMS condenser microphone (MEMS CMIC) are given in Reference [13].

**Figure A2:**
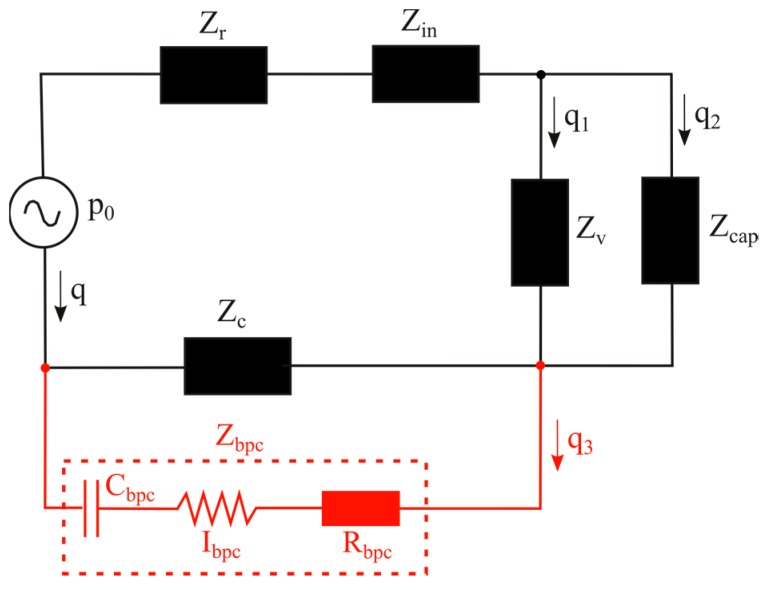
Equivalent electrical circuit representing the lumped element model (LEM) of the MEMS condenser microphone (MEMS CMIC) adapted with pneumatic access for static pressure equalization (SPEQ) required as soon the microphone is sealed (red highlighted circuit). The black part of the circuit represents a standard LEM of an MEMS CMIC, as given in References [25,26]. *Z_r_*: acoustic radiation impedance; *Z_in_*: inlet channel (pressure port); *Z_cap_*: diaphragm, back plate and electrical impedance of the capacitor; *Z_v_*: vent channel(s) for SPEQ; and *Z_c_*: back cavity.

A LEM representing an MEMS MIC sealed with one PD forms a circuit which can be reduced to four lumped elements (Figure 4a). One element describes the mass and stiffness of the PD (*Z_PD_*) and its interaction with the surrounding fluid (reactive and dissipative, *Z_R_*). The second and third element describe the pneumatic tubing system between the PD and the MEMS MIC which adds loss, inertia (*Z_PT,_*_1_) and compliance (*Z_PT,_*_2_) to the system. The last element represents the unsealed MEMS MIC *Z_MEMS_*, which is adapted with an SPEQ system that is required as soon the MEMS MIC is sealed (Appendix A, Figure A1, Figure A2, Table A1). In addition, the pressure port of an unsealed MEMS MIC and its interaction with the surroundings (semi-open channel radiating into the far-field) are neglected in the sealed case. All lumped elements, except *Z_PT,_*_2_ that is connected in parallel to *Z_PT,_*_1_ and *Z_MEMS_*, are connected in series.

A circuit including more than one PD (Figure 4b) cannot be solved using simple rules of calculation for serial and parallel circuits, as applied for an unsealed MEMS MIC or one sealed with only one PD (Figure 4a).

(1) $$Zq=p\;Z=\left[\begin{array}{ccc} Z_{11} & \cdots & Z_{1m}\\ \vdots & \ddots & \vdots \\ Z_{n1} & \cdots & Z_{nm} \end{array}\right]q=\left[\begin{array}{c} q_{1}\\ \vdots \\ q_{n} \end{array}\right]p=\left[\begin{array}{c} p_{1}\\ \vdots \\ p_{n} \end{array}\right]$$

For more complicated electrical circuits, a system of linear equations must be solved. Such a system expressed in matrix form is given in Equation (1), where ***Z*** denotes the impedance matrix, ***q*** is the vector including the volume flows in the system and ***p*** represents the solution vector containing the input sound pressure *p_o_*. The matrix ***Z*** and the vector ***p*** follow from Kirchhoff’s laws applied to the equivalent electrical circuit [24]. The corresponding components for a microphone sealed with four PDs and the calculations for all lumped elements are given in Appendix A (Figure A1, Table A2, Table A3, Table A4 and Table A5). Finally, the system is solved for ***q*** = ***Z***^−1^***p***. To simulate a device with less than four PDs, the unused branches of the circuit are disabled by choosing a very high value for the impedance of the corresponding PD (***Z****_1_* to ***Z****_4_*).

(2) $$S_{v}=\left|\frac{V_{o}}{p_{o}}\right|=\left|\frac{V_{b}u_{dia,MEMS}}{i\omega d_{g}}\right|$$

The open-circuit voltage sensitivity *S_v_* of the sealed microphone is calculated using Equation (2), where *V_o_* denotes the open-circuit voltage at the MEMS CMICs electrical output, *p_o_* is the incoming sound pressure, *V_b_* represents the bias voltage, *ω* is the angular frequency, *d_g_* denotes the gap between the diaphragm and the back plate of the MEMS CMIC and *u_dia,MEMS_* represents the velocity of the vibrating diaphragm. 

(3) $$q_{13}=q_{5}+q_{6}+q_{7}+q_{8}$$

The velocity *u_dia,MEMS_* is derived from *q*_13_, the volume flow through the MEMS CMIC (Equation (3)) and the LEM circuit of the unsealed MEMS CMIC, as given in References [25,26]. All input parameters required for the LEM of the MEMS CMIC, such as *V_b_*, *d_g_* and all other geometrical dimensions, are stated in Reference [13]. They were estimated from optical inspection of the device and iterative parameter adaptation to obtain a satisfactory agreement between the frequency response stated in the datasheet and that calculated using the LEM.

#### 2.3.2. Theoretical Model of the SPEQ System

**Figure 5:**
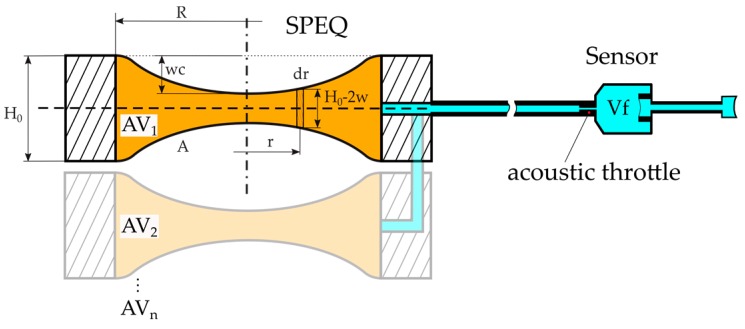
Schematic drawing of the static pressure equalization (SPEQ) system of an intracochlear acoustic receiver (ICAR) that is based on an adaptive volume (AV) consisting of a stack of *n* diaphragm pairs suspended on a rigid support structure.

A simple theoretical model describing the behaviour of the SPEQ system based on the adaptive volume (AV) was created to demonstrate the feasibility of this design concept. The main design assessment criterium is the size of the AV required to cover the specified operating pressure range from 700 mbar to 1200 mbar. The model considers a flat and a corrugated diaphragm pair suspended on a rigid support structure and forming the AV (Figure 5). A tube of length *L* pneumatically interconnects the AV with the receiver unit. An acoustic throttle at the interface between tube and receiver prevents that AV induced sound waves above 200 Hz (lower limiting frequency) are entering the back chamber of the receiver. The receiver and the tube are considered the fixed volume *V_f_* of the system. During volume adaptation, both diaphragms need to deflect up to 10 times their thickness, where non-linear effects cause mechanical stiffening of the diaphragm. Corrugated diaphragms show longer linear travel than flat diaphragms and hence, should broaden the operating range of the SPEQ system [27]. In case the AV is restricted by the lateral dimension but not by the height, *n* diaphragm pairs may add up to a stack to achieve the required volume size (Figure 5).

(4) $$p_{amb}A=K\left(w_{c}\right)w_{c}+p_{i}\left(w_{c}\right)A$$

Equation (4) represents the governing equation describing the equilibrium of forces acting on the diaphragm of the AV. The ambient pressure is depicted by *p_amb_*; the surface area, the centre deflection and the mechanical stiffness of the diaphragm are represented by *A*, *w_c_* and *K*, respectively; and the internal pressure of the system is denoted by *p_i_*.

(5) $$K\left(w_{c}\right)=\frac{p_{m}\left(w_{c}\right)}{w_{c}}A$$

(6) $$\frac{p_{m}R^{4}}{Et^{4}}=\frac{5.33}{1-v^{2}}\left(\frac{w_{c}}{t}\right)+\frac{2.83}{1-v^{2}}\left(\frac{w_{c}^{3}}{t^{3}}\right)$$

The stiffness *K* is described as the mechanical force *p_m_A* required to deflect the diaphragm by *w_c_* (Equation (5)). This characteristic quantity is obtained by numerically solving Equation (6), which represents the relationship between the mechanical load *p_m_* and *w_c_* for a flat diaphragm without intrinsic stress but taking into account large deflections [28]. A similar relationship for a corrugated, circular diaphragm can be found in the literature [29]. 

(7) $$p_{i}\left(w_{c}\right)=\;\frac{p_{i,0}V_{i,0}}{V_{i}\left(w_{c}\right)}$$

(8) $$V_{i,0}=nAH_{0}+V_{f}$$

The internal pressure *p_i_* at a certain *w_c_* is calculated from the ideal gas law defining an initial state at *p_i_*_,0_ and the initial volume of the system *V_i_*_,0_ (Equation (7)). *V_i_*_,0_ is the sum of the fixed volume *V_f_* and the stack of *n* adaptive volumes at zero *w_c_* (Equation (8)). 

(9) $$V_{i}\left(w_{c}\right)=n2\overset{R}{\underset{0}{\int }}r\pi \left(H_{0}-2w\left(r\right)\right)dr+V_{f}$$

(10) $$w\left(r\right)=w_{c}{\left(1-{\left(\frac{r}{R}\right)}^{2}\right)}^{2}$$

Because the *w_c_* can reach values higher than 10% of *H*_0_, the curvature of a deflected diaphragm is considered in the calculation of AV at a certain *w_c_* (Equations (9) and (10)). 

From Equation (4), it is obvious that a full equilibrium between the ambient and internal pressure is only achievable at zero *w_c_*. As soon as the diaphragm starts to deflect, *p_i_* will start to deviate from pressure equilibrium and will reach maximum deviation at the highest *w_c_*. This is the operating point of the ICAR with a maximum loss in sensitivity. During the design of the SPEQ system, a parameter study was performed with the aim of finding the size and geometry of an AV that can provide SPEQ within the specified pressure range and a tolerable loss in sensitivity (Section 3.2.2).

### 2.4. Experimental Verification of the Receiver Unit of the ICAR

The ICAR receiver unit was experimentally verified using a simple prototype of an MEMS MIC sealed with multiple PDs. The sensor performance was determined in air in an audiometer and in water in an anechoic water tank. Tests in air are favourable due to the smaller experimental complexity compared with tests conducted in water. On the other hand, the validity is only given at frequencies well below resonant operation of the sensor. In this regime, the sensor is purely stiffness driven and the operating medium (air or water) has no influence on the ICAR’s sensitivity. As soon as reactive forces start to dominate the sensor behaviour (resonant operation), this assumption does not hold anymore. In a microphone operating in air at frequencies below 10 kHz, the most dominant reactive part of the system stems from the inlet channel (pressure port), which, in combination with the back cavity of the microphone, forms a Helmholtz resonator. In water, the PD sealing the microphone experiences high inertia and hence, a resonant operation below 10 kHz. In this case, the PD’s resonance characterizes the sensing behaviour (sensitivity and bandwidth) of the transducer within the upper operating frequency range. To study the sensor’s behaviour within the resonant operating regime of the transducer, experimental tests in an anechoic water tank were conducted, whereas tests in air were performed to obtain the low-frequency behaviour.

#### 2.4.1. Multi-Diaphragm ICAR Prototype

**Figure 6:**
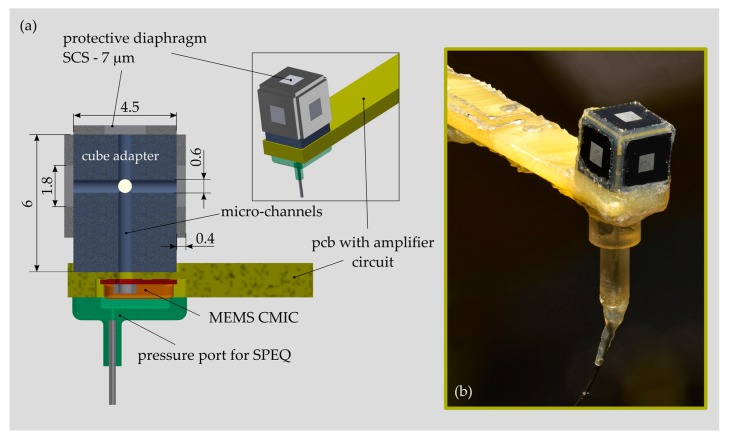
(**a**) Design of a multi-diaphragm intracochlear acoustic receiver (ICAR) prototype used for experimental validation of a lumped element model (LEM) representing the present packaging concept for an MEMS MIC; assembly drawing with components and dimensions and (**b**) photograph of prototype.

The acoustic receiver was built on the basis of a MEMS CMIC (ADMP504, Analog Devices, Inc., Norwood, MA, USA) that was sealed using a single-crystal silicon (SCS) diaphragm available from a former project [30] (Figure 6). The square SCS diaphragm was 1.8 mm large, 7 μm thick and suspended on a 4.5 mm large and 0.4 mm high SCS support. The MEMS CMIC was recessed within the front part of a printed circuit board (PCB) carrying an amplifier and a power supply circuitry. The multi-diaphragm design concept was realized by mounting up to four PDs onto a cube-like structure made of plastic and attached to the pressure port of the MEMS CMIC. Micro-channels with a diameter of 0.6 mm were integrated into the cube adapter to pneumatically interconnect all PDs and the MEMS CMIC. In order to test sensor configurations with less than four PDs, the spare ones were removed and the micro-channel was tightly sealed using a rigid closure and silicone adhesive. In order to allow for SPEQ between the airtight sealed sensor interior and the surroundings, a tiny orifice was punched (diameter < 0.1 mm) into the metallic housing of the MEMS CMIC. A pneumatic fitting was connected to the orifice to enable the installation of a hose for active back pressure control, as required during testing in water.

The first prototype of the ICAR enabled testing of the multi-diaphragm sensor concept and validation of the LEM of the ICAR but it did not cover similar dimensions of relevant parts of the inner ear microphone, such as the size of the PD(s) and the volume of the pneumatic tubing connecting the PD(s) and the MEMS MIC. The larger size of the sensor compared to the final design, implied an uncertainty of LEM validation and did not allow sound pressure measurements in the cochlea of human cadaveric temporal bones. Such investigations are of high importance for ICAR development. Because of these unmet needs, a prototype with a smaller form-factor was developed. This sensor is not implantable and does not feature a multi-diaphragm design but it did further validate the LEM and is frequently used for investigations of the cochlea hydrodynamics. A detailed description of the design, fabrication and testing of that sensor is published in References [13,15].

#### 2.4.2. Sensor Testing in Air

The frequency response of the ICAR prototype in air was measured using a clinical audiometer (Affinity 2.0, Interacoustics Inc., Middelfart, Denmark) as an acoustic test chamber and a data acquisition unit (USB-6215, National Instruments, Austin, TX, USA) for recording of the microphone’s output signal. The audiometer allows microphone testing (stimulation) up to a frequency of 10 kHz and a sound pressure level of 90 dB SPL. A reference microphone positioned close to the sensor under investigation allows closed-loop control of the desired sound pressure level. The software Affinity HIT440 (Interacoustics, Inc., Middelfart, Denmark) was used to generate pure tones at one-third octave-band frequencies between 125 Hz and 10 kHz and 90 dB SPL. Each pure tone was played for several seconds before switching to the next frequency. During that time, the analogue signal of the MEMS CMIC was acquired during 1 s and with 100 kHz sample rate for each frequency step using the data acquisition unit and LabVIEW (National Instruments, Austin, TX, USA). Processing of the raw data was conducted in Matlab (The MathWorks, Inc., Natick, MA, USA). First, a band-pass filter (50 Hz to 20 kHz passband) was applied to the raw data; then, the RMS value was determined; and finally, the sensitivity of the sensor in dB re V/Pa was calculated. 

Characterization of the self-noise of the sealed microphone was conducted in a small anechoic chamber (anechoic egg, Type 4222, Brüel & Kjaer, Nairom, Denmark) that provides a test environment well decoupled from surrounding noise and mechanical vibrations. During measurement, the power supply of the ICAR prototype was switched on and a stepped frequency sweep performed according to the procedure mentioned above but without applying acoustic stimulation. During data processing in Matlab, a band pass filter with one-third octave bands and centre frequencies corresponding to the frequencies of the sweep was first applied on the acquired data before calculating the RMS value. Averaging of the RMS values from 20 subsequent measurements was performed. The noise data were expressed either as one-third octave EIN pressure considering the measured frequency response of the ICAR prototype or as a noise voltage in one-third octaves. 

#### 2.4.3. Sensor Testing in Water

**Figure 7:**
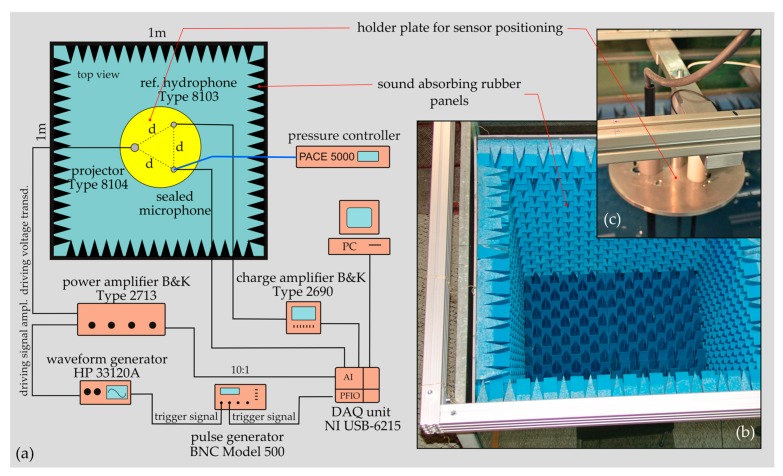
(**a**) Schematic illustration of the experimental setup used for intracochlear acoustic receiver (ICAR) prototype testing in an anechoic water tank using the comparison calibration technique. (**b**) Tank lined with sound absorbing rubber panels (the absorber panels covering the open water surface are not shown in the picture). (**c**) System for sensor fixation and positioning in the water tank

Sensor testing in water was conducted in a large water tank of 1 × 1 × 1 m^3^ (Figure 7). In order to approximate free-field conditions inside the tank, the interior was lined with 100 mm thick sound-absorbing rubber panels of type SA-J100 (SUASIS Underwater Systems, Kocaeli, Turkey). The free surface of the water tank was covered with four floating absorber panels mounted on a polystyrene support. 

For ICAR testing, the so-called comparison calibration technique was employed, which is based on a comparison of the unknown sensor with a well-calibrated reference acoustic receiver. As a sound source, a piezoelectric hydrophone (Type 8104, Brüel & Kjaer, Nairom, Denmark) and as a reference receiver, the hydrophone (Type 8103, Brüel & Kjaer, Nairom, Denmark) were used. The reference receiver and the sealed microphone were placed at two different positions which were equidistant to the projector. The separation distance between the transducers of 0.1 m was chosen so that cross interference between the devices and the measurement uncertainty due to spatial variations in the pressure field were minimized. Each transducer was mounted at the end of a 1 m long carbon fibre tube and precisely arranged in a triangular configuration using an aluminium holder plate (Figure 7c). The whole sensor unit was positioned in the centre of the tank (immersion depth 0.5 m). A power amplifier (Type 2713, Brüel & Kjaer, Nairom, Denmark) and a waveform generator (HP 33120, Hewlett Packard, Palo Alto, CA, USA) were used to drive the piezoelectric projector at high voltage levels (<100 V_rms_). For signal averaging, a pulse generator (BNC Model 500, Berkeley Nucleonics Corp., San Rafael, CA, USA) was used as a trigger source to synchronize the sound source and the data acquisition (DAQ) unit (Figure 7a). For each frequency step, the gain of the power amplifier was adjusted to a predefined value to obtain sound pressure amplitudes of approximately 1 Pa at the reference hydrophone location. Because of the decreasing transmitting efficiency of a piezoelectric projector with a decreasing frequency, an amplitude of 1 Pa could not be attained for frequencies below 800 Hz. The output of the reference hydrophone was routed through a charge conditioning amplifier (Type 2690, Brüel & Kjaer, Nairom, Denmark) and acquired together with the output signal of the ICAR prototype and the driving signal of the projector (voltage divider 10:1). For data acquisition, a DAQ unit (USB-6215, National Instruments, Austin, TX, USA) controlled by Matlab was used. The data were sampled with 83 kHz for 0.15 s and averaged over 30 subsequent measurements per frequency step. Data were collected in one-third octave band steps between 125 Hz and 10 kHz. A pressure controller (PACE 5000, GE Druck Ltd., Leicester, UK) was used to adapt the internal pressure of the hydrophone submerged in the water tank to the corresponding hydrostatic pressure. The hydrostatic pressure was determined by measuring the depth of immersion of the sensor *H* and by the hydrostatic equation *ρgH*, where *ρ* denotes the density of water and *g* is the gravitational acceleration. The back pressure was adjusted to the specific value prior the measurement and kept constant within ±20 Pa during measurement.

The anechoic water tank is not sufficiently soundproofed to allow reliable characterization of the self-noise of the ICAR prototype operating in water. Instead, the anechoic egg and the procedure similar to the noise measurements conducted in air (Section 2.4.2) but with the ICAR immersed in a small container filled with water were used for noise measurements.

### 2.5. Sensor Design and Fabrication

**Figure 8:**
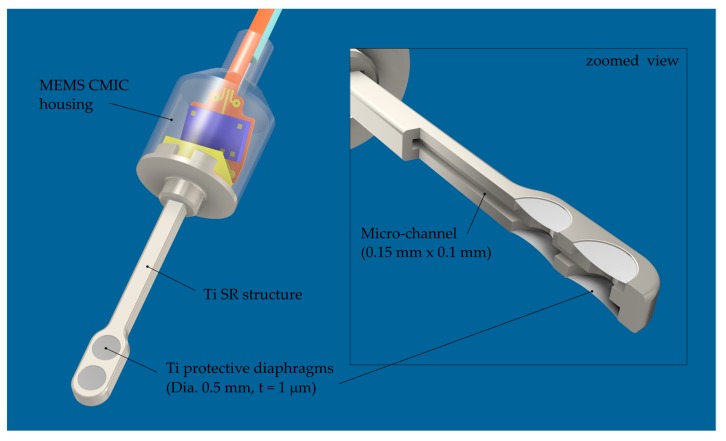
Drawing of the intracochlear acoustic receiver (ICAR) made from titanium and equipped with four protective diaphragms within the tip region of the sound receptor.

The main constraints affecting the packaging structure design of the implantable ICAR were (1) restricted space conditions in the middle ear cavity and the inner ear and (2) meeting the required sensing performance for a TICI. The resulting design of the ICAR is illustrated in Figure 8. The SPEQ system, as an integral part of the packaging structure, is not shown in the figure. It is discussed and illustrated separately in Section 2.5.3. 

#### 2.5.1. Sound Receptor (SR)

The SR forms a 5 mm long tube-like structure that is widened within the tip region, where several PDs are arranged in a pairwise configuration on the top and bottom of the SR. The PDs are all pneumatically interconnected with the MEMS MIC through a micro-channel with a square cross section (0.15 mm × 0.1 mm). LEM calculations have shown that four 0.6 mm large and 1 μm thick circular Ti diaphragms are needed to meet the requirements for the sensing performance (Section 3.2.1). A rectangular cross-section (0.35 mm × 0.3 mm) of the SR structure is justified by the chosen fabrication process, which is briefly described below. 

**Figure 9:**
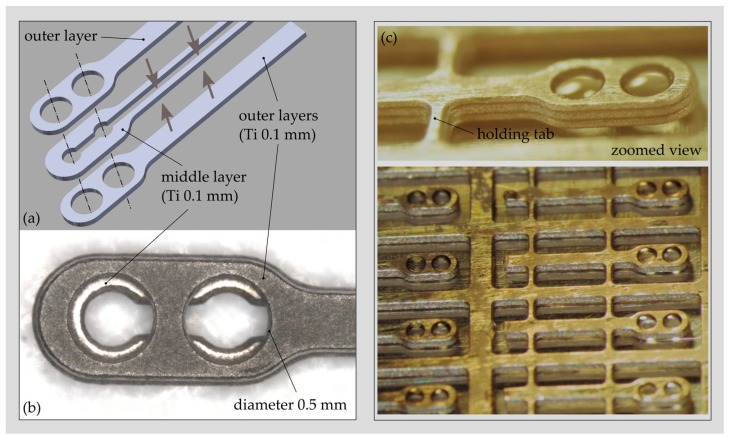
(**a**). CAD drawing showing the three-layer design of the sound receptor (SR) structure, including the geometry of the individual layers. (**b**) The tip region of the bare SR structure fabricated by photo-chemical etching and diffusion bonding. (**c**) SR structures after cavity filling with QPAC 40.

The fabrication process is based on two commercial fabrication technologies—photo chemical etching (PCE) and diffusion bonding (DB)—to fabricate the bare SR structure first and in a second step to complement it with the PD’s using DC magnetron sputtering of Ti. Due to process restrictions imposed by the PCE process, the SR is made from three 0.1 mm thick CP Grade 2 titanium sheets that are first structured by PCE and then joined by DB (Figure 9a,b). Bonds obtained with DB are classified as being long-term hermetic. The SR structures are fabricated in a panel configuration with a size of 100 mm × 100 mm, obtaining more than 200 SR units per substrate. 

A novel fabrication process was developed for PD fabrication on the bare SR structures. The process relies on a low-temperature decomposable polymer (QPAC 40, Empowers Materials Inc., New Castle, DE, USA), which serves as sacrificial material for the subsequent thin film deposition process of Ti. The QPAC solution is applied to the SR structure using a pneumatic dispensing system. A flat or slightly curved mirror-like surface remains after curing at 150 °C (Figure 9c). After thin film deposition of Ti (low-power DC magnetron sputtering), the QPAC is completely decomposed into CO_2_ and water between 250 °C and 350 °C. 

So far, intact 1 μm thick PDs could be fabricated using this novel fabrication process. Currently, an extensive study is ongoing, with the aim of optimizing the deposition process to minimize the intrinsic stress (compliance) and the number of pores (hermeticity) within the diaphragm.

#### 2.5.2. Microphone Housing

**Figure 10:**
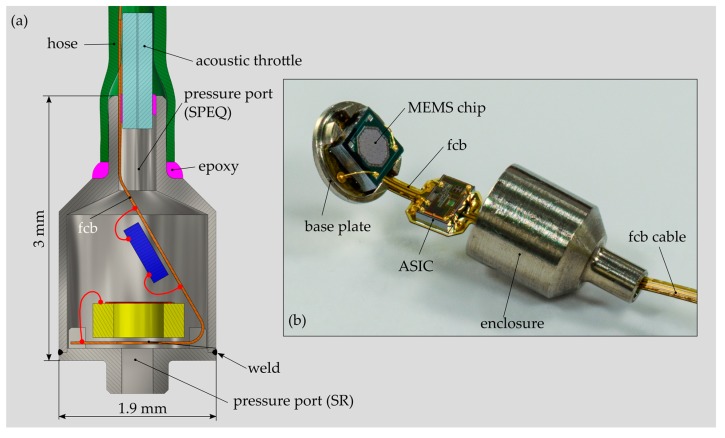
(**a**) Computer-aided-design (CAD) drawing of the MEMS condenser microphone (MEMS CMIC) installed in customized Ti housing. The drawing shows the non-planar arrangement of the MEMS chip and ASIC that allows a housing geometry with an optimal form-factor to be used for ICAR insertion into the inner ear (adapted from Reference [15]) and (**b**) the assembled flexural circuit board (FCB) before installation of the microphone enclosure.

The MEMS CMIC (ADMP803, InvenSense Inc., San Jose, CA, USA) is installed in a cylindrical titanium housing with a diameter of 1.9 mm and an overall height of 3 mm (Figure 10a). A cylindrical geometry was determined as the optimal one for insertion through the facial recess and the placement of the microphone housing in the middle ear cavity. The two parts of the housing—the base plate and the enclosure—were fabricated by classical micro-machining technology (Figure 10b). After microphone installation, both parts were micro-laser welded to obtain a robust and hermetic joint. In order to minimize the diameter of the housing, the ASIC is arranged above the MEMS chip facing the back plate of the CMIC. A thin flexural circuit board (FCB) provides the mechanical and electrical interface between the MEMS chip and the ASIC. In addition, the FCB integrates a 100 mm long flexible cable that simplifies the electrical interface between the microphone and the external amplifier unit. A 1.5 mm long fused-silica tube with a capillary lumen of 20 microns diameter was inserted and gas-tight sealed in the pressure port channel. The capillary tube serves as an acoustic throttle for SPEQ with high-pass filter characteristics and a low cut-off frequency smaller than 100 Hz. The microphone housing and the ground plane of the FCB are electrically interconnected for EMI shielding. For testing a printed circuit board (PCB) including an amplifier unit (gain = 50, type OP ADA4004, Analog Devices, Norwood, MA, USA) and a power supply circuitry was interconnected with the MEMS CMIC over the integrated FCB cable [15].

The first packaging trial confirmed the feasibility of this novel solution for MEMS MIC packaging in a cylindrical housing. However, noise measurements (procedure cf. Section 2.4.2) revealed an equivalent input noise that was averaged over the considered frequency range, 10 dB higher compared to the reference device in commercial packaging. It is presumed that the altered self-noise may result from an inappropriate electrical interface between FCB and ASIC. This issue shall carefully be considered during the next packaging trial. 

#### 2.5.3. SPEQ System

**Figure 11:**
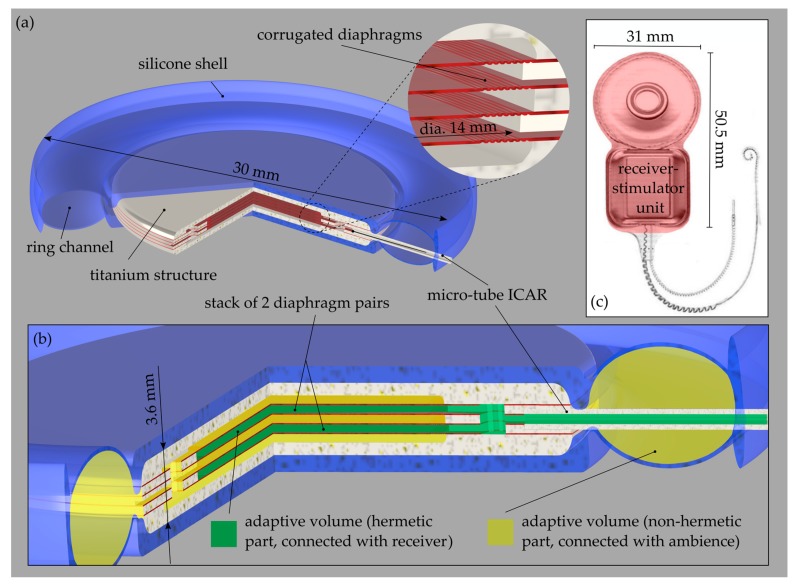
(**a**) CAD drawings (three quarter section view) of the static pressure equalization (SPEQ) system which is based on an adaptive volume (AV) structure integrated in the stimulator-receiver unit of the cochlear implant (CI) system. (**b**) Zoomed view onto the interior of the AV structure. The colours indicate the two parts of the pneumatic system. Green is the hermetic part connected to the receiver and yellow the non-hermetic part connected to the ambience over the ring channel (see legend). (**c**) The CI system (Type Nucleus CI512 Cochlear Ltd., Sydney, Australia) with relevant dimensions. Red colour indicates the receiver-stimulator unit of the CI, which is foreseen for the integration of the AV of the SPEQ system.

The receiver-stimulator unit of the CI situated in the mastoid (behind the ear) promises to be a location for the structure carrying the adaptive volume (AV) of the SPEQ system. Such a design configuration is illustrated in Figure 11 and discussed in the subsequent paragraph. However, the feasibility of such a system has to be first verified in an extensive study considering system integration capabilities, cross-interferences with the receiver and patient comfort with the implant. 

The AV is integrated into a titanium structure (TS) with coin-like geometry with a diameter of 19 mm and a thickness of 3.6 mm. A stack of two diaphragm pairs that are pneumatically interconnected and well protected by the TS should provide the required performance for SPEQ. The individual diaphragms have a corrugated design, a diameter of 14 mm and a thickness of 9 μm (Figure 11b, green). They are separated and rigidly clamped between three 0.2 mm thick Ti plates forming the TS. In order to prevent damage of the diaphragm due to excessive loading, the maximum diaphragm deflection is limited to 10 times the diaphragm thickness. Correspondingly, the total volume of the system can vary from 49 mm^3^ to 94 mm^3^, with a fixed part of 8 mm^3^ formed by the receiver unit and the micro-tube interconnecting the receiver and the SPEQ system. Several radially oriented micro-channels are integrated into the TS to pressurize the exterior of the AV (Figure 11b, yellow). To prevent blockage of the vent channels by growing tissue or liquids, the TS is embedded into a silicone shell which exhibits a ring channel circumferentially arranged around the TS. The ring channel forms an additional AV, which is not hermetic but should provide enough protection against the mentioned contaminants (Figure 11b, yellow). The ring must be highly compliant to minimize losses in the transfer of ambient pressure to the AV. A Ti micro-tube pneumatically interconnects the hermetic part of the AV with the ICAR. The AV (metal part) should be batch-fabricated from multiple Ti sheets of different thickness that are first structured by photo-chemical etching and finally joined by compression bonding to obtain a hermetic structure. The implementation of the proposed design will first start after successful verification of the theoretical model and the system integration feasibility. 

## 3. Results

### 3.1. Experimental LEM Validation

**Figure 12:**
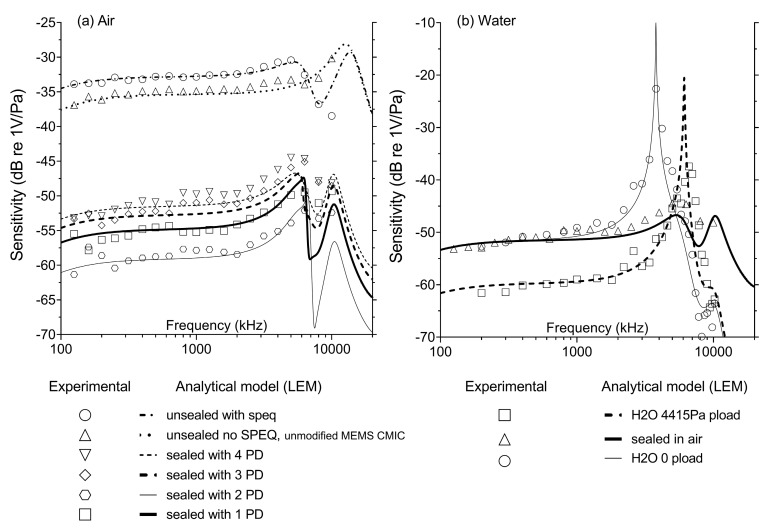
(**a**) Frequency response of the ADMP504 microphone sealed with one, two, three and four protective diaphragms (PDs) and operating in air. Markers: experiment, lines: analytical model (LEM). For comparison, the frequency response of the unsealed device equipped with and without (unmodified) a pressure port for static pressure equalization (SPEQ) is also shown. (**b**) Frequency response of the MEMS condenser microphone (MEMS CMIC) sealed with four PDs operating in water and in air. In addition, the frequency response of a sealed microphone that experiences the full hydrostatic pressure at a 0.5 m insertion depth is shown (p_load_ = 4415 Pa).

The LEM representing an MEMS CMIC sealed with multiple PDs and equipped with a pressure port for SPEQ was validated by sensor calibration in air and in water using the first prototype of the ICAR (Section 2.4.1). Tests in air comprised sensor configurations equipped with one, two, three and four PDs and the unsealed microphone with and without an additional pressure port for SPEQ (Figure 12a). The frequency response of the unsealed MEMS CMIC in its original packaging was measured to provide reference data for fine adjustment of the input parameters of the corresponding LEM. Validation of the LEM in water was conducted using a sensor configuration with four PDs only (Figure 12b). 

As shown in Figure 12a, the unsealed microphone equipped with a pressure port for SPEQ leads to an altered frequency response compared to the unsealed device without an SPEQ system. The additional volume formed by the pressure fitting of the SPEQ system increases the sensitivity well below the resonance frequency by 3 dB but also causes an additional resonance (Helmholtz resonator) at frequencies below the resonance of the unmodified MEMS CMIC. The LEM adapted with the SPEQ system (Appendix A, Figure A1, Figure A2, Table A1) seems to accurately predict the modified sensing behaviour of the MEMS CMIC at frequencies below 10 kHz. Since no experimental data were available for frequencies above 10 kHz, the second resonance peak, which appears in the model data above 10 kHz, could not be confirmed by the experiment. A satisfactory agreement between the model and experiment was also achieved for the microphone sealed with different numbers of PDs. The sensitivity of the device well below resonance operation can be predicted with an accuracy lower than ±3 dB. This accuracy is within the measurement uncertainty of the audiometer. Within the resonance operation, the model provides a sensitivity that shows larger variation with frequency, which is a behaviour that cannot be fully reproduced by the experiment. The deviation might be explained by the increasing sensitivity to sound pressure gradients of the relatively large ICAR prototype in comparison with the wavelength of air-borne sound at higher frequencies. However, the model can accurately predict the point of the first resonance peak. As a result of the increased volume enclosed by the PDs and the MEMS CMIC diaphragm, the point of resonance shifts to lower frequencies with an increasing number of PDs (Helmholtz resonator). In addition, a larger intermediate volume results in a larger compliance and hence, a lower volume flow velocity, which is transferred from the PDs to the diaphragm of the MEMS CMIC. This effect becomes apparent if the increase in sensitivity well below the resonance operation is compared for a two and four PD sensor configuration in reference to the one PD design. While the sensitivity increases by almost 5 dB if going from one to two PDs, there is no significant benefit in performance if using more thanfour PDs

Figure 12b clearly illustrates that the ICAR prototype operating in water shows the same sensitivity as in air within the flat frequency response range but a different behaviour within the resonance operation. The pronounced resonance peak in water stems from the resonance of the PD experiencing a much higher load in water than in air. The LEM representing an MEMS CMIC sealed with four PDs and operating in water can handle both operation regimes very well. The resonance of a PD vibrating in water and having a compliance that is required for an ICAR of a TICI would limit the sensor’s bandwidth to frequencies well below the level of a TICI. The multi-diaphragm SR design counters this trade-off behaviour by using multiple smaller PDs with a higher resonance frequency instead of one large PD that would fulfil the compliance but not the band-width requirements. To prevent any static pressure loading of the PD during the experiments in the water tank, the internal pressure of the sensor was adapted to the hydrostatic pressure at the corresponding insertion depth (4415 Pa). If no pressure equalization was performed, the sensitivity of the microphone was reduced and the resonance frequency shifted to higher frequencies. This behaviour was also accurately reproduced by the LEM. 

### 3.2. Theoretical Verification of the Sensor Design

#### 3.2.1. Predicted ICAR Sensing Performance

**Figure 13:**
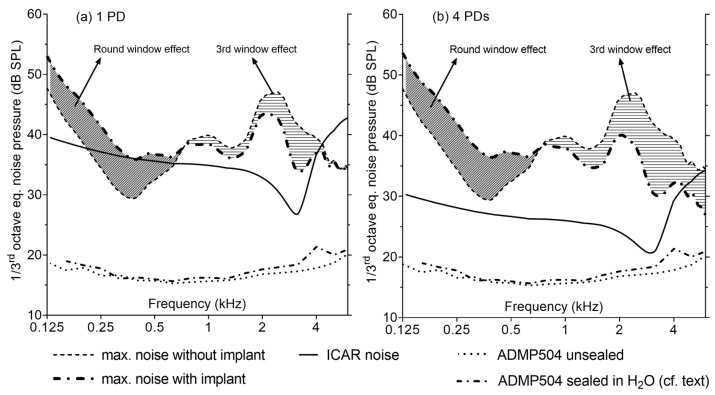
(**a**) Predicted (LEM) one-third octave equivalent noise pressure of the proposed intracochlear acoustic receiver (ICAR) with one and (**b**) four protective diaphragms (PDs) and equipped with the ADMP504 MEMS microphone. The sensor performance is compared with the noise requirements for an implant inserted in the scala tympani (ST). The dashed areas reflect potential deviations in intracochlear sound pressure caused by round window (RW) reinforcement (doubled RW stiffness) and sensor input impedance (third window effect). The experimentally determined noise characteristics of the ADMP504 microphone in an unsealed and sealed configuration (operating in water) are also shown. For the purpose of comparison, the frequency response of the unsealed microphone is used for the noise calculation of both sensor configurations.

An LEM study was conducted to define all relevant dimensions of an ICAR that meets the performance and size requirements defined for a TICI system. From the frequency response of the simulated device and the self-noise measured on the ICAR prototype in water (Section 2.4.3), the sound pressure level was determined, which is equivalent to the input noise expressed in one-third octaves (black solid line in Figure 13). The pneumatic system connecting the PD(s) and the MEMS CMIC of the ICAR prototype and the liquid operating medium have no significant influence on the self-noise of the receiver (thin dashed dotted line in Figure 13). The equivalent input noise (EIN) level of the unsealed ADMP504 (Analog Devices, Inc., Norwood, MA, USA) microphone measured in the anechoic egg (Section 2.4.2) is taken as the base line (thin dotted line in in Figure 13). For comparison of the EIN of the two different sensor configurations (sealed and unsealed), the frequency response of the unsealed microphone is considered for the calculation of the EIN of the sealed microphone as well. Another noise study using the ICAR prototype with similar SR size as the implantable ICAR [13] confirmed that even SR tubes with a length of up to 18 mm and a channel diameter of 0.15 mm do not significantly increase the self-noise of the sealed receiver. 

Figure 13a shows the sensing performance of an ICAR with dimensions as described in Section 2.5 but instrumented with only one titanium PD and Figure 13b reports results for a multi-diaphragm sensor with four PDs. The maximum noise level that can be tolerated for an ICAR situated in the scala tympani (ST) corresponds to the 30 dB HL line plus the pressure gain of the outer and middle ear considered at sensor’s location (dashed line in Figure 1 and Figure 13). CIs that are inserted into the cochlea through the round window (RW) cause reinforcement of the RW and hence, an increase in sound pressure in the basal part of the ST at frequencies below 1 kHz. The effect of RW reinforcement (doubled RW stiffness) on the sound pressure in the ST was approximated using a simple LEM of the cochlea adopted from Nakajima et al., 2009 [8]. The increase in sound pressure in the ST after RW reinforcement is confirmed by the work of Xiying et al., 2018 [31]. 

**Figure A3:**
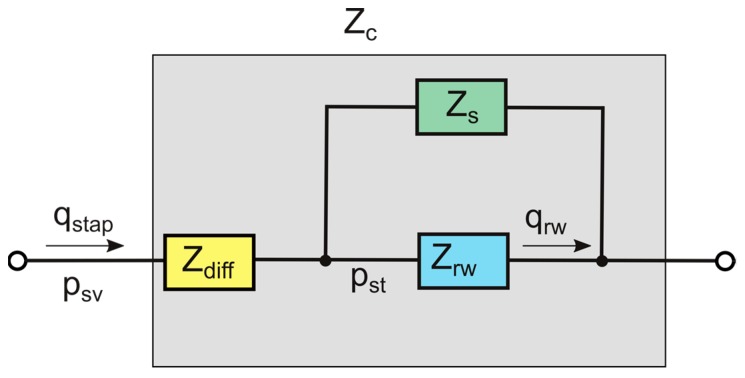
Equivalent electrical circuit representing a lumped element model (LEM) of the cochlea with an intracochlear acoustic receiver (ICAR) implanted in the scala tympani. *Z_c_*: cochlear input impedance, *Z_diff_*: differential impedance, *Z_rw_*: round window impedance, *Z_s_*: sensor impedance, *p_sv_*: vestibuli sound pressure, *p_st_*: tympani sound pressure, *q_stap_*: volume velocity of the stapes foot plate and *q_rw_*: volume velocity of the round window.

It is also expected that an ICAR with similar input impedance to the cochlea impedance will act as a pressure release valve. To estimate the effect of the ICAR on the sound pressure in the ST, the LEM of the cochlea was complemented with the acoustic impedance representing the ICAR (Appendix B, Figure A3, Table A6). The tolerable noise level of an ICAR if both effects are considered is indicated by a dashed dotted line in Figure 13. At frequencies below 700 Hz, the RW effect dominates and causes an increase in sound pressure up to 7 dB. Above that frequency, a pressure drop due to the pressure release effect may be expected. The drop in sound pressure reaches its maximum at the resonance operation of the sensor (lowest input impedance). So far, no experimental data are available to validate the theoretical model describing the pressure release effect caused by the sensor. 

Figure 13 illustrates that an ICAR with a single PD cannot meet the requirements for the sensing performance at frequencies below 700 Hz if the effect of RW reinforcement is not considered. Using the multi-diaphragm design with four PDs, the noise level stays below the noise limit over almost the whole considered frequency range (150 Hz–6 kHz). Above 5 kHz, the noise limit is exceeded only if the decrease in sound pressure due to the inserted sensor is considered.

#### 3.2.2. Predicted SPEQ System Performance

**Figure 14:**
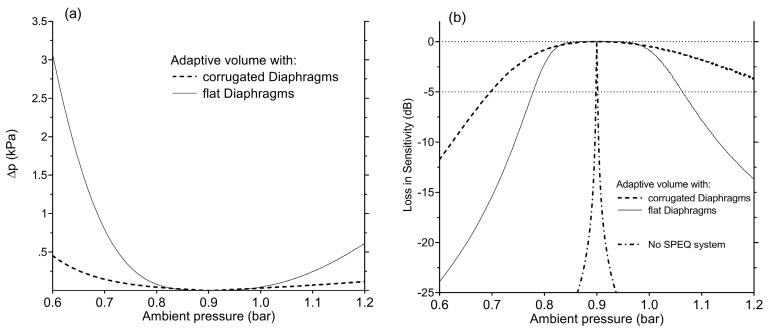
(**a**) Deviation from equilibrium between the internal and external pressure as a function of the ambient pressure between 600 and 1200 mbar. Considered is an adaptive volume (AV) containing two diaphragm pairs equipped with flat or corrugated diaphragms. The initial internal pressure is set to 900 mbar. (**b**) Loss in the intracochlear acoustic receiver’s (ICAR’s) sensitivity *S_m_* with ambient pressure variations relative to 900 mbar. For comparison, the drop in sensitivity of an ICAR without static pressure equalization (SPEQ) is shown

The performance of an SPEQ system which is based on a passive adaptive volume (AV) is verified by theoretical means (Section 2.3.2). Figure 14 depicts the performance of an SPEQ system with dimensions that should allow system integration into the receiver-stimulator unit of the CI (Section 2.5.3). The corresponding diaphragms of the AV are 14 mm large, 9 μm thick and are either flat or corrugated. They form a stack of two diaphragm pairs. For an initial internal pressure of 900 mbar and 600 mbar or 1200 mbar ambient pressure (maximum volume adaptation), the diaphragm deflection does not exceed 10 times the diaphragm thickness. Ambient pressure variations within these limits include most daily life situations such as weather changes, swimming and air travel. The SPEQ performance in Figure 14 is expressed in two ways: Figure 14a shows the remaining deviation from pressure equilibrium (ideal SPEQ system) and Figure 14b illustrates the resulting loss in mechanical sensitivity *S_m_* of an ICAR equipped with such an SPEQ system. The considered operating pressure range of the SPEQ is between 600 and 1200 mbar. The PD of the ICAR considered for this study is 0.5 mm large, 1 μm thick and made from titanium. 

The benefit of an SPEQ system equipped with corrugated instead of flat diaphragms is clearly seen in the performance data. Due to the lower stiffness of a corrugated diaphragm at large deflections (x >> t), the deviation from equilibrium is decreased by more than a factor of 5 compared to the flat design. Without an SPEQ system, the sensitivity drops by more than 20 dB already for small ambient pressure variations (<20 mbar). Using an SPEQ system with a corrugated diaphragm design, the loss in sensitivity can be kept below 5 dB over the specified pressure operating range (700–1200 mbar). 

## 4. Summary and Discussion

A packaging concept for commercial MEMS MICs was developed that enables the use of this high-end microphone technology as an ICAR of a TICI system. The packaging structure made from titanium was designed to provide (1) long-term hermetic sealing of the delicate and non-biocompatible parts of the MEMS MIC and (2) sensing of the sound pressure in the perilymph of the human inner ear. Further important design criteria were associated with the need for simple surgical intervention and high sensing performance. Well-defined protective Ti diaphragms were integrated into the packaging structure to enable the sound energy to pass through the hermetic structure with minimum loss. The geometry of the packaging structure was designed such that the sound pressure in the scala tympani (ST) could be recorded with the MEMS MIC located in the larger middle ear cavity. 

To minimize the loss in sensing performance caused by ambient pressure variations, an SPEQ system based on volume adaptation was developed that could adapt the internal pressure of the packaging structure to the prevailing ambient pressure without an open pneumatic interface with the surroundings. Using a theoretical model of the SPEQ system, an adaptive volume was designed that could limit the drop in ICAR sensitivity due to ambient pressure variations in a range between 700 and 1200 mbar to 5 dB. In everyday life, typically only weather induced ambient pressure variations well below +/−100 mbar occur. In such conditions, SPEQ system related changes in ICAR’s performance should not be noticeable by the user. The size of the corresponding adaptive volume structure enables a potential installation in the receiver-stimulator unit of the CI. 

In order to specify the design parameters of the ICAR that meets requirements for a TICI system, an LEM was created that incorporates the packaging concept with multiple PDs. Experimental validation of the LEM was conducted in water in an anechoic water tank and in air in a clinical audiometer. The sensor used for these validation tests was built based on an MEMS CMIC that was sealed with up to four PDs made from single-crystal silicon. The tests confirmed a prediction accuracy of the LEM smaller than ±3 dB over almost the whole frequency operating range of the ICAR. This prediction accuracy was only exceeded within resonance operation of the sensor characterized by larger variations in sensitivity with frequency. A parameter study conducted using the validated LEM revealed that at least four titanium PDs with a diameter of 0.6 mm and a thickness of 1 μm are needed to meet the required sensing performance for an ICAR implanted in the ST of the human inner ear. 

Considering these geometrical constraints and different aspects and limitations of Ti fabrication, a packaging structure for the MEMS MIC was designed that meets all the requirements defined for an ICAR (long-term biocompatibility and hermeticity and optimum form factor). The design foresees a titanium enclosure consisting of three main parts: the sound receptor, the microphone housing and the SPEQ system. They are all fabricated independently and finally assembled by micro-laser welding. A cylindrical microphone housing with a minimum cross section was evaluated as the most suitable geometry for sensor insertion and placement in the middle ear cavity. The minimum cross section of the housing was achieved with a stacked arrangement of the MEMS chip and the ASIC. A thin, highly flexible circuit board (FCB) provides the electrical and mechanical interface between the two parts of the MEMS CMIC. The first assembly trials confirmed the feasibility of this novel encapsulation technology for MEMS MICs. 

A novel fabrication process has been presented that allows the integration of 1 micron thick and sub-millimetre large Ti diaphragms on a tube-like Ti support structure representing the sound receptor of the ICAR. The process separates the fabrication of the 3D support structure and the Ti PDs, which enables the use of commercial Ti fabrication technology (photo-chemical etching, diffusion bonding) for manufacturing the support structure. The PDs are fabricated based on DC magnetron sputtering of Ti on a low-temperature decomposable polymer that serves as sacrificial material supporting the Ti film during the deposition process and finally is removed by thermal treatment at 350 °C. So far, critical fabrication steps have been identified and their feasibility verified. Currently, process optimization is going on, with the aim of obtaining Ti PDs with small intrinsic stress and providing long-term hermetic sealing. 

As soon as this has been accomplished, chronic in vivo experiments in sheep will follow, which shall verify the sensing performance of the ICAR in a living subject considering fibrosis growth and cross interference with body and skin contact noise. Further development of the SPEQ system will be conducted in parallel. It will include the fabrication of a large-scale model of the SPEQ design to confirm the theoretical model and a full-scale dummy model to evaluate and optimize system integration.

## 5. Patents

Walraevens, J. Cochlear Implant Electrode Array Including Receptor and Sensor. US Patent 20150126900A1, 7 May 2015.

Walraevens, J. Internal Pressure Management System. US Patent 20150367130A1, 24 December 2015.

## Appendix A

A microphone sealed with a protective diaphragm requires a system for static pressure equalization (SPEQ) to maintain constant sensitivity with varying ambient pressure and temperature. The SPEQ system, as implemented in the multi-diaphragm prototype, is shown in Figure A1.

It is composed of an orifice in the metallic housing of the MEMS condenser microphone (MEMS CMIC), which is enclosed by a pneumatic fitting forming a cavity and a micro-channel. In order to account for the SPEQ system in the lumped element model (LEM), a corresponding impedance *Z_bpc_* is connected in parallel with the impedance representing the back cavity of the unsealed MEMS CMIC *Z_c_* (Figure A2). Table A1 lists only the formula and parameters required to calculate *Z_bpc_*. The LEM of the unsealed MEMS CMIC is not covered here but can be found in References [25,26].

**Table A1:** Lumped parameter model for the acoustic impedance *Z_bpc_* of the system for static pressure equalization (SPEQ) [kg/s/m^4^], [23].

$R_{bpc}=\frac{8l\mu }{\pi r^{4}}$	*R_bpc_*: acoustic resistance of the vent channel [kg/s/m^4^]	(A1)
$I_{bpc}=\frac{4}{3}\frac{\rho l}{\pi r^{2}}$	*I_bpc_*: acoustic inertance of the vent channel [kg/m^4^]	(A2)
$C_{bpc}=\frac{V_{ic}}{\rho c^{2}}\;V_{ic}=l_{ic}b_{ic}h_{ic}$	*C_bpc_*: acoustic compliance of the cavity ***V_ic_*** [m^4^s^2^/kg].	(A3)
$Z_{bpc}=\;R_{bpc}+i\omega I_{bpc}+\frac{1}{i\omega C_{bpc}}$		

*l* = 0.4 mm: length of the vent channel (orifice); *r* = 0.07 mm: radius of the orifice; length *l_ic_* = 3 mm, width *b_ic_* = 1.5 mm and height *hic* = 0.5 mm of the cavity; *V_ic_*, *c*: speed of sound in air [m/s]; *μ*: dynamic viscosity of air [Pa s]; and *ρ*: density of air [kg/m^3^].

**Table A2:** Definition of matrix ***Z*** and vector ***p*** representing a system of linear equations, which describes the lumped element model (LEM) of the microphone sealed with four protective diaphragms.

***Z*** =	**nm**	**1**	**2**	**3**	**4**	**5**	**6**	**7**	**8**
	1	*Z*_1_	−*Z*_2_	0	0	*Z*_5_	−*Z*_6_	0	0
	2	*Z*_1_	0	−*Z*_3_	0	*Z*_5_	0	−*Z*_7_	0
	3	*Z*_1_	0	0	−*Z*_4_	*Z*_5_	0	0	−*Z*_8_
	4	−Z_9_	0	0	0	(*Z*_5_ + Z_9_ + *Z*_*MEMS*_)	*Z*_*MEMS*_	*Z*_*MEMS*_	*Z*_*MEMS*_
	5	0	−*Z*_10_	0	0	*Z*_*MEMS*_	(*Z*_6_ + *Z*_10_ + *Z*_*MEMS*_)	*Z*_*MEMS*_	*Z*_*MEMS*_
	6	0	0	−*Z*_11_	0	*Z*_*MEMS*_	*Z*_*MEMS*_	(*Z*_7_ + *Z*_11_ + *Z*_*MEMS*_)	*Z*_*MEMS*_
	7	0	0	0	−*Z*_12_	*Z*_*MEMS*_	*Z*_*MEMS*_	*Z*_*MEMS*_	(Z_8_ + Z_12_ + Z_*MEMS*_)
	8	(*Z*_1_ + Z_9_)	0	0	0	−Z_9_	0	0	0
***p*** =		0	0	0	0	0	0	0	*p*_0_

**Table A3:** Lumped parameter model for the acoustic radiation impedance *Z_r_* and acoustic impedance of a protective diaphragm *Z_p,d_*.

**Model for the acoustic radiation impedance *Z_r_* [kg/s/m^4^], [23]:** Transducer represents a piston at the end of a long tube (piston constitutes the side of a box). Model requirements: *k**·r_p_ <* ½ ≥ radiation is omnidirectional (for 10 kHz *r_p_* < 3 mm in air and < 12 mm in water).		
$R_{r}=\frac{\pi }{4}\rho ck^{2}r_{p}^{4}$	*R_r_*: radiation resistance [kg/s]	(A4)
$X_{r}=1.9\omega \rho r_{p}^{3}$	*X_r_*: acoustic radiation mass (reactance) [kg/s]	(A5)
$Z_{r}=\frac{1}{A_{d,p}^{2}}\left(R_{r}+iX_{r}\right)$	*Z_r_*: acoustic radiation impedance [kg/s/m^4^]	(A6)
**Model for the acoustic impedance of the protective, square diaphragm *Z_d,p_* [kg/s/m^4^]**		
$p=\frac{E}{\left(1-v^{2}\right)}{\left(\frac{t_{p}}{a_{p}}\right)}^{4}\left[67.2\left(\frac{w_{c}}{t_{p}}\right)+25.28{\left(\frac{w_{c}}{t_{p}}\right)}^{3}\right]$	Relationship between pressure load *p* [Pa] and centre deflection *w_c_* [m] (large deflections) of a square diaphragm with no intrinsic stress, [32].	(A7)
$C_{d,p}=\frac{1}{A_{d,p}}\frac{dw_{c}}{dp}=\frac{1}{A_{d,p}}S_{m,d}$	*C_d,p_* [m/N], *S_m,d_* [m^3^/N]: mechanical compliance and sensitivity of the protective diaphragm.	(A8)
$m_{d,p}=\frac{1}{C_{d,p}{\left(2\pi f_{r}\right)}^{2}}$	*m_d,p_*: equivalent mass of the diaphragm [kg]	(A9)
fr=fr0[1+1.18(wctp)2]12	*f_r_*: resonance frequency of a square diaphragm (fundamental mode) for any static deflections [Hz], [33].	(A10)
fr0=35.992πap2[Etp212ρs(1−v2)]12	*f_r_*_0_: resonance frequency of a square diaphragm at zero deflection [Hz], [34].	(A11)
$Z_{d,p}=\frac{1}{A_{d,p}^{2}}\left(i\omega m_{d,p}+\frac{1}{i\omega C_{d,p}}\right)$	Acoustic impedance of the protective, square diaphragm *Z_d,p_* [kg/s/m^4^]	(A12)
$Z_{1,2,3,4}=Z_{r}+Z_{d,p}$		(A13)

*k*: angular wave number [rad/m], *c*: speed of sound [m/s], *ω*: angular frequency [rad/s], *r_p_*: radius of the vibrating surface [m] (for square protective diaphragm *A_dp_*; *r_p_* = (*A_dp_/π*)^−1/2^, *a_p_*: side length of a square diaphragm [m], *t_p_*: diaphragm thickness [m], *ρ*: fluid density [kg/m^3^], *ρ_s_*: density of the diaphragm material [kg/m^3^], *E*: Young’s modulus [N/m^2^] and *v*: Poisson’s ratio [–].

**Table A4:** Lumped parameter model for the acoustic impedance of the pneumatic system connecting the protective and the MEMS microphone’s (MEMS MIC’s) diaphragm [kg/s/m^4^], [23].

$m_{ae}=\frac{4}{3}\frac{\rho l_{ps}}{A_{ps}}$	*m_ae_*: acoustic inertance of the connecting pipe by considering a parabolic flow [kg/m^4^].	(A14)
$R_{a}=8\frac{\mu l_{ps}}{\pi r_{ps}^{4}}$	*R_a_*: acoustic resistance of a pipe with a very small diameter [kg/s/m^4^]	(A15)
$Z_{ps}=R_{a}+i\omega m_{ae}$	*Z_ps_*: dissipative and reactive component of the impedance representing the pneumatic system [kg/s/m^4^].	(A16)
$C_{ps}=\frac{V_{ps}}{\rho c^{2}}$	*C_ps_*: compliance of the pneumatic system [kg/s/m^4^]	(A17)
$V_{ps}=A_{d,p}h_{c}+A_{ps}l_{ps}+V_{add}$	*V_ps_*: volume of the pneumatic system [m^3^]	(A18)
$V_{add}=4\left(r_{ps}^{2}\pi l_{ps}\right)+\left(r_{ps,add}^{2}\pi l_{ps,add}\right)+V_{MEMS}$	*V_add_*: additional volume of the pneumatic system independent of the number of protective diaphragm (PDs).	(A19)
$Z_{5,6,7,8}=Z_{ps}$		(A20)
$Z_{9,10,11,12}=\frac{1}{i\omega C_{ps}}$		(A21)

*A_ps_*: cross section of the pipe [m^2^], ***c***: speed of sound in air [m/s] and *μ*: dynamic viscosity of air [Pa s]. *ρ*: density of air [kg/m^3^].

**Table A5:** Lumped parameter model for the acoustic impedance of the MEMS microphone (MEMS MIC) *Z_MEMS_* [kg/s/m^4^], [23].

$Z_{tot,\;mic\_us}$	*Z_tot,mic_us_*: total acoustic impedance of an unsealed MEMS MIC without considering the pressure port and the interaction between the inlet channel and the surroundings (acoustic radiation) [kg/s/m^4^], calculated based on References [13,25,26]	
$Z_{ps,add}$	*Z_ps,add_*: additional reactive and resistive impedance of the pneumatic system that is independent of the number of protective diaphragms [kg/s/m^4^], calculated based on Equations (A11)–(A13) with *r_ps,add_* and *l_ps,add_* (cf. Figure A1)	
$Z_{MEMS}=Z_{tot,\;mic\_us}+Z_{ps,add}$		(A22)

## Appendix B

The cochlear input impedance *Z_c_* is defined as the ratio between the vestibuli pressure *p_sv_* and the stapes volume velocity *q_stap_*. According to Nakajima et al. 2008 [8], *Z_c_* can be described by a serial circuit of the differential impedance *Z_diff_ = (p_sv_* − *p_st_)/q_stap_* and the round window impedance *Z_rw_ = p_st_/q_rw_*, where *p_st_* denotes the pressure in the scala tympani and *q_rw_* is the volume velocity of the round window. The acoustic receiver is represented by one lumped element describing the total acoustic sensor impedance *Z_s_*. If the sensor is implanted in the scala tympani, it bypasses the round window and hence, *Z_s_* is connected in parallel to *Z_rw_*.

**Table A6:** Analytical approach for estimating the loss in tympani pressure after the implantation of an intracochlear acoustic receiver (ICAR) in the scala tympani.

$P_{s1}=P_{s2}$	Assumption: constant sound power to the cochlea, Subscripts 1 and 2: before and after implantation	(A23)
$\frac{p_{sv1}}{p_{sv2}}=\sqrt{\frac{Z_{c1}}{Zc_{2}}}$	Follows from Equation (A23) and $\;P_{s}=p_{sv}q_{stap}$ and $p_{sv}=Z_{c}q_{stap}$	(A24)
$p_{st1}=p_{sv1}\frac{Z_{rw}}{Z_{c1}}$ and $p_{st2}=p_{sv2}\frac{Z_{rw,s}}{Z_{c2}}$ with $\;Z_{rw,s}=\frac{Z_{rw}Z_{s}}{Z_{rw}+Z_{s}}$	Follows from equivalent electrical circuit (Figure A3)	(A25)
$r=\frac{p_{st2}}{p_{st1}}=\frac{Z_{rw,s}}{Z_{rw}}\sqrt{\frac{Z_{c1}}{Z_{c2}}}$ with $Z_{c1}=Z_{diff}+Z_{rw}$ and $\;Z_{c2}=Z_{diff}+Z_{rw,s}$	Follows from Equations (A24) and (A25)	(A26)

To estimate the loss in tympani pressure *p_st_* caused by the implant, a constant sound power *P_s_* (independent of *Z_c_*) is assumed at the input of the cochlea (Equation (A23)). The loss in tympani pressure *r*, which is defined as the ratio between ***p_st2_*** and ***p_st1_*** (A26), is calculated using the Equations (A23)–(A25). The impedances *Z_diff_* and *Z_rw_* are taken from the work of Nakajima et al. [8]. The data were derived from simultaneous sound pressure measurements in the basal part of the scala tympani and vestibuli and Laser Doppler Velocimetry (LDV) on the stapes footplate of several fresh human temporal bones. The input impedance of the sensor *Z_s_* was determined using the LEM of the ICAR sealed with multiple protective diaphragms (Section 2.3.1).

The influence of round window impedance modification on the tympani pressure was estimated using Equation (A26) without considering the sensor (*Z_s_*) in the calculation of the ratio *r* and an LEM of *Z_rw_*, as stated in Nakajima et al., 2008 [8]. The inertia (i.e., inductance) and viscous dissipation (i.e., resistance). For *Z_rw_* modification only the compliance wasvaried, whereas the viscous losses and inertia were kept constant.
